# Dispersive growth and laser-induced rippling of large-area singlelayer MoS_2_ nanosheets by CVD on c-plane sapphire substrate

**DOI:** 10.1038/srep11756

**Published:** 2015-06-29

**Authors:** Hongfei Liu, Dongzhi Chi

**Affiliations:** 1 Institute of Materials Research and Engineering (IMRE), A*STAR (Agency for Science, Technology and Research), 3 Research Link, Singapore 117602, Singapore

## Abstract

Vapor-phase growth of large-area two-dimensional (2D) MoS_2_ nanosheets via reactions of sulfur with MoO_3_ precursors vaporized and transferred from powder sources onto a target substrate has been rapidly progressing. Recent studies revealed that the growth yield of high quality singlelayer (SL) MoS_2_ is essentially controlled by quite a few parameters including the temperature, the pressure, the amount/weight of loaded source precursors, and the cleanup of old precursors. Here, we report a dispersive growth method where a shadow mask is encapsulated on the substrate to ‘*indirectly*’ supply the source precursors onto the laterally advancing growth front at elevated temperatures. With this method, we have grown large-area (up to millimeters) SL-MoS_2_ nanosheets with a collective in-plane orientation on c-plane sapphire substrates. Regular ripples (~1 nm in height and ~50 nm in period) have been induced by laser scanning into the SL-MoS_2_ nanosheets. The MoS_2_ ripples easily initiate at the grain boundaries and extend along the atomic steps of the substrate. Such laser-induced ripple structures can be fundamental materials for studying their effects, which have been predicted to be significant but hitherto not evidenced, on the electronic, mechanical, and transport properties of SL-MoS_2_.

The bottom-up growth of large-area singlelayer (SL), i.e., two-dimensional (2D), materials such as graphene and group-VI transition metal dichalcogenides (e.g., MoS_2_) has been the subject of extensive studies over the last few years, driven by the potentially wide range of applications in the fields of nanoelectronics^1^,^2^,^3^,^4^,^5^, nanophotonics^6^,^7^, and energy harvesting^8^,^9^. Since 2008 mass-production of continuous graphene films by chemical vapor deposition (CVD) on polycrystalline metals (e.g., Ni and Cu) via thermal decomposition of hydrocarbons directly on the surface or surface segregation of carbon atoms from a metastable carbon-metal solid solution upon cooling has been well developed^1^,^10^,^11^. As a comparison, the CVD growth of large-area SL-MoS_2_ nanosheets via simply sulfurizing pre-deposited Mo/MoO_3_ films or direct reactions of S atoms with MoO_3_ precursors vaporized and transferred from their powder sources onto a target substrate is still under development although a number of devices have recently been demonstrated based on the MoS_2_ atomic layers grown by these methods^12^,^13^,^14^,^15^,^16^.

The challenge in CVD growth of large-area SL-MoS_2_ nanosheet is multifold. In general, the thermal-vapor sulfurization method can generate large-area and macro-scale uniform MoS_2_ atomic layers^16^,^17^,^18^,^19^. However, to increase the adatom mobility so as to grow high quality MoS_2_ atomic layers with larger grain sizes, the growth temperature must be elevated, which in turn results in an undesired surface evaporation^12^. The balance between the adatom mobility and the surface evaporation leads to the limited grain sizes and/or the limited yields of SL films^12^. On the other hand, the CVD growth of 2D MoS_2_ via direct reactions on the substrate is essentially controlled by quite a few parameters, such as the growth temperature, the chamber pressure, the source-substrate setup, the amount of loaded source precursors, and the cleanup of old precursors^20^,^21^,^22^,^23^,^24^.

Recent experimental improvements in CVD growth of MoS_2_ atomic layers nevertheless make the studies of their microstructures, e.g., grains and grain boundaries, and device performances possible^21^,^24^. In comparison, ripple structures, which have been theoretically predicted to have remarkable effects on the electronic and mechanical properties of SL-MoS_2_^25^,^26^, have so far only been observed and investigated by transmission-electron microscopy in suspended SL-MoS_2_ nanosheets prepared by exfoliation^27^,^28^. These spontaneously formed SL-MoS_2_ ripples are about 0.6–1.0 nm in height^27^,^28^, analogues to those of graphene^29^. Although it has been proposed that periodic ripple structures can be fabricated by putting or growing SL nanosheets on a wavy substrate^26^,^30^, e.g., for bandgap engineering^26^, no experimental work on generating SL-MoS_2_ ripple structures has been reported in the literature.

Here, we report an experimental observation for regular ripple structures induced by laser illuminations/scanning into high-quality SL-MoS_2_ nanosheets. The SL-MoS_2_ nanosheets up to millimeter in dimensions were grown by CVD at elevated temperatures on c-plane sapphire substrates. Unlike previous work^14^,^24^, a shadow mask of Si or quartz, physically encapsulated on the substrate, is introduced to realize the ‘*dispersive*’ growth^31^, i.e., the source species are dispersively, rather than ‘*directly*’, supplied onto the laterally advancing growth front under the shadow mask during the CVD growth at elevated temperatures. Raman scattering and photoluminescence (PL) spectroscopy as well as atomic-force microscopy (AFM) have been employed to compare the SL-MoS_2_ nanosheets and their rippling induced by laser illuminations. Both the dispersive CVD growth method and the laser-induced rippling may have important consequences in experimental and theoretical studies of SL-MoS_2_ and other 2D materials.

## Results

**Figure 1:**
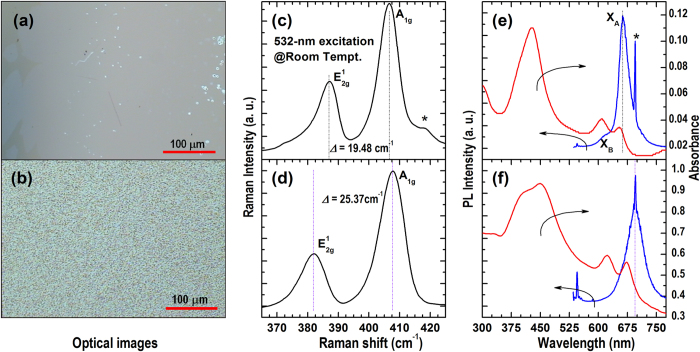
Optical images, Raman scattering, photoluminescence (PL), and absorbance spectra of MoS_2_ atomic layers grown by CVD (using S and MoO_3_ powders as the reaction precursors) on c-plane sapphire substrate at 950 °C. (**a**) Optical image, (**c**) Raman spectrum, and (**e**) PL and absorbance of a MoS_2_ sample grown by the dispersive method. (**b**) Optical image, (**d**) Raman spectrum, and (**f**) PL and absorbance of a MoS_2_ sample grown by the conventional direct method. The asterisk indicates the emission from the sapphire substrate.

Figure 1 shows a comparison between the MoS_2_ atomic layers grown by CVD on c-plane sapphire substrates using a direct and a dispersive method, respectively. MoO_3_ and S powders, the same as previous work^20^, were used as the precursors in the CVD growth; however, we have introduced a shadow mask (i.e., a clean Si or quartz disk with the surface roughness of ~5 μm as measured by AFM) to partly encapsulate the surface of the substrate to generate the dispersive growth (see SI, Fig. S1). The growth parameters are, otherwise, the same for the directly and the dispersively grown MoS_2_ samples. Figures 1a,b show the optical images of MoS_2_ grown by the dispersive and the direct method, respectively. Triangle and hexagonal MoS_2_ grains (see SI, Fig. S2) are also observed in the directly grown sample at the areas a bit far away from the MoO_3_ source. The Raman, PL, and absorbance spectra in Figs 1c,e clearly show that high-quality SL-MoS_2_ is obtained with the dispersive growth method^32^,^33^,^34^. As a comparison, the increased A_1g_-E_2g_^1^ difference and the shifted (to longer wavelengths) PL emission and exciton resonance absorption peaks (see Figs 1d,f) indicate that thicker MoS_2_ atomic layers are resulted in the direct growth^32^,^33^,^34^. The apparently shifted but still strong and narrow PL emissions of the MoS_2_ atomic layers indicate their high crystal quality due to the elevated growth temperature (950 °C) employed in this study.

A closer look at the SL-MoS_2_ nanosheets in Fig. 1a reveals some linear structures in the center areas; their directions are either parallel or 60°-rotated with one another (see SI, Fig. S3a). These linear structures are most likely caused by the grain boundaries^21^,^24^, where the unsaturated bonds can be the nucleation sites for the incorporation of disorders and/or contaminations. This observation implies that the growth of MoS_2_ on the c-plane sapphire substrate has a collective in-plane orientation in large-area. The collective in-plane orientation is also evidenced by lowering the dispersive growth temperature from 950 to 750 °C (see SI, Fig. S3b). The lowered growth temperature leads to an incomplete grain coalescence that shows the collective in-plane orientation of the triangular grains manifested by their paralleled edges. We also found that one edge of the individual triangular grain is parallel to the [11–20] axis of sapphire (i.e., the direction of the primary wafer flat). This relation suggests an in-plane rotation of 30° between MoS_2_ and c-plane sapphire, i.e., MoS_2_[10-10]//Al_2_O_3_[11**-**20]. In this light, the collective in-plane orientation of MoS_2_ is most likely defined by the 60°-rotation symmetric crystalline substrate. Fast Raman and PL mappings (100 × 100 μm^2^) with a mapping step of 4 μm revealed that the A_1g_-E_2g_^1^ frequency differences of the SL-MoS_2_ nanosheet are strongly correlated with its PL emission wavelengths (see SI, Fig. S4). 95% of the 625 experimental data fall in the range of Δ = 19.5 ± 0.5 cm^−1^ and λ_PL_ = 661.0 ± 2.0 nm. A few sets of Raman-PL mappings from 0.5-mm-separated areas revealed no apparent variations in Δ or λ_PL_. These results, together with the larger-area collective in-plane orientation of MoS_2_, indicate that large-area high-quality SL-MoS_2_ nanosheets are obtained on c-plane sapphire substrates by CVD using the dispersive growth method.

**Figure 2:**
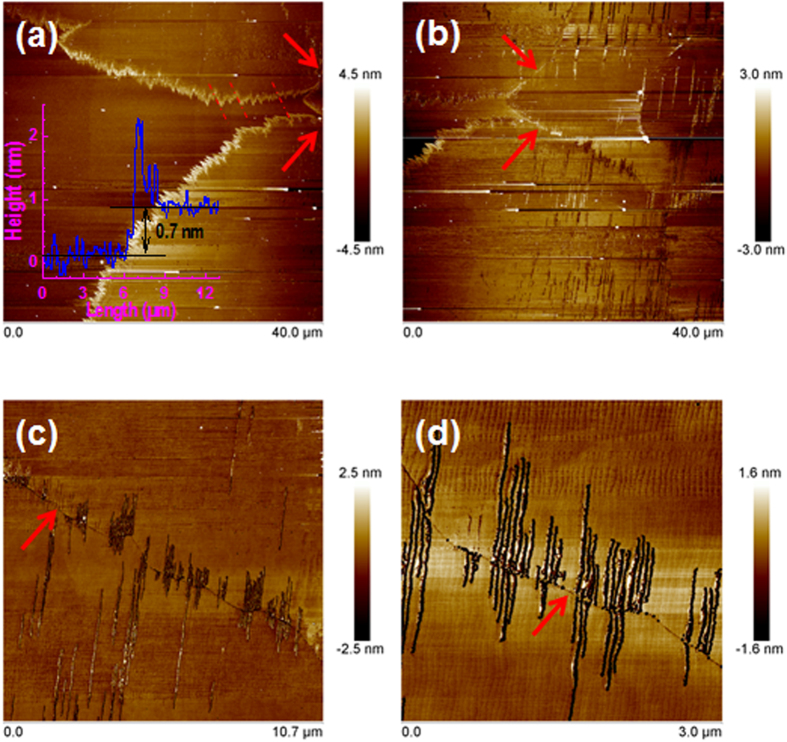
AFM images taken from the MoS_2_ atomic layers grown by CVD using the dispersive method. Images (**a**) and (**b**) are in large area, showing the grain edges and the grain boundaries as indicated by the arrows. Images (**c**) and (**d**) are zoom-in of the grain boundaries. The inset in (**a**) is the height profile across the grain edge of MoS_2_ on sapphire while the dashed lines indicate the parallel micro-edges of the saw-like grain edge, they are also parallel with respect to one another between the top and bottom grains.

Figure 2 shows the AFM images taken from the area where the fast Raman and PL mappings were carried out. The inset in Fig. 2a is a height profile, which reveals that the thickness of the MoS_2_ nanosheet is about 0.7 nm, corresponding to the thickness of SL-MoS_2_. Also seen in Figs 2a,b is that the grain edges consist of zigzag structures and they are more or less parallel to one other as indicated by the dashed lines in Fig. 2a. This observation indicates that the large SL-MoS_2_ grain is a single crystal. Grain boundaries, indicated by the arrows, are seen in Figs 2b–d. MoS_2_ ripples across the grain boundary are clearly seen; they extended following the atomic steps of the substrate as seen in Fig. 2d. Since the AFM images were taken after the fast Raman and PL mappings, together with the laser-induced ripples (see later discussions), we believe that these ripples were induced by the laser illuminations during the fast Raman and PL mappings and the presence of grain boundary made the MoS_2_ rippling feasible.

**Figure 3:**
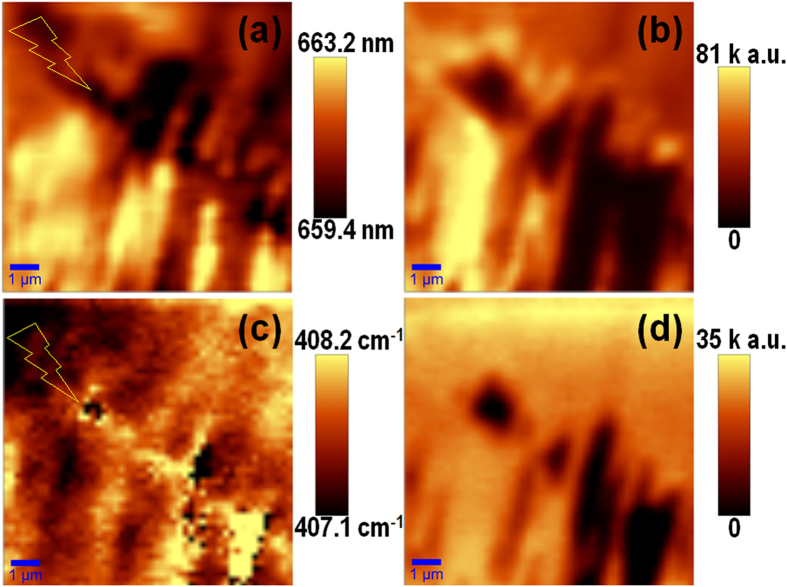
PL and Raman mappings with a step of 0.4 μm from a grain boundary area. (**a**) Wavelength of the PL emission peak, (**b**) Intensity of the PL emission, (**c**) Raman shift of A_1g_, and (**d**) Intensity of A_1g_. The lightning bolts in (**a**) and (**c**) indicate the grain boundary, extending from left-up to right-bottom, which is in fact the same one seen in Figures 3(c–d).

To verify the laser-induced SL-MoS_2_ rippling we have carried out a further round of Raman and PL mappings for a square of 10 × 10 μm^2^ with the step size reduced to 0.4 μm. Figures 3a,b show the emission wavelength and peak intensity of the PL mapping, respectively. Likewise, Figs 3c,d present the A_1g_ frequency and intensity of the Raman mapping, respectively. The strong correlation in the contrast distributions between Figs 3a–d indicate that the Raman and PL mappings were precisely carried out at the same area of the SL-MoS_2_ sample. A comparison between Figs 3a,c as well as the AFM image in Fig. 2c shows that the grain boundary, as indicated by the lightning bolts, resulted in blue shifts to both the PL emissions and the A_1g_ vibrations. It should be noted that the E_2g_^1^ mode (results not shown for the sake of brevity) also exhibits a blue shift at the grain boundary. The observed blue shifts in the PL emission and Raman phonons of SL-MoS_2_ at the grain boundaries are consistent with those reported by van der Zande *et al.*^24^; unfortunately, the origin of the blue shifts is unclear at this stage since both the strain and doping effects might present at the grain boundaries.

**Figure 4 f4:**
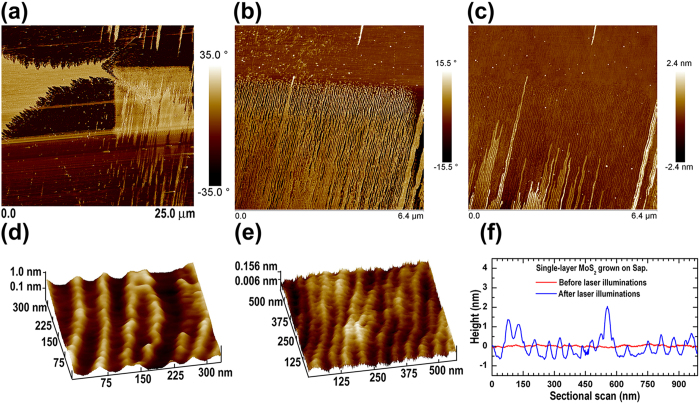
AFM images showing the laser-induced rippling of the SL-MoS_2_ nanosheet. (**a**) AFM phase image, clearly showing the surface changes, i.e., the 10 × 10 μm^2^ square in the right side of the image, (**b**) Phase image and (**c**) Height image of an AFM scanning at the square edge with an increased magnification, (**d**) AFM image focused on the rippled area, i.e., inside the laser illuminated square, (**e**) AFM image focused on the non-rippled area, i.e., outside the laser illuminated square, (**f**) Height profiles collected from the areas in and out the square, i.e., before and after the laser illuminations.

Figure 4a shows an AFM phase image taken from the area where the slow Raman and PL mappings were carried out. One sees a 10 × 10 μm^2^ square, having a sharp contrast from its surroundings, induced by the laser illuminations. AFM imaging with a larger magnification at the top edge of the laser illuminated square was further carried out; the phase and height images are shown in Figs 4b,c, respectively. Figures 4d,e show the fine AFM comparisons taken from the areas in and out the square, respectively. Likewise, Fig. 4f shows the height profiles collected from the AFM images taken in and out the square. These results, together with those seen in Fig. 2, provide clear-cut evidence that regular ripple structures have been created in the SL-MoS_2_ nanosheets by the laser illuminations during the slow Raman and PL mappings. The comparisons between Figs 4d,e as well as those in Fig. 4f revealed that the MoS_2_ ripples arranged themselves along the atomic steps of the c-plane sapphire substrate. A closer look at the images in Figs 4a[Fig f4]b as well as the optical contrast evolutions (see SI, Fig. S5) confirmed that the initiation of SL-MoS_2_ ripples is much easier at the grain boundaries.

The average ripple period is ~50 nm (see Figs 4d,f). The much smaller surface undulation (<0.2 nm) of the as-grown SL-MoS_2_ in Figs 4e,f suggests the conformal growth of MoS_2_ on the c-plane sapphire substrate. Another piece of evidence for the conformal growth of 2D-MoS_2_ nanosheets on c-plane sapphire is shown in SI (Fig. S6), where one clearly sees the continuity of the surface atomic steps of sapphire across the grain edges of MoS_2_. Due to the conformal growth, the surface undulation of the as-grown SL-MoS_2_ is exactly following the surface steps of the c-plane sapphire (see Figs 4e,f and SI, Fig. S7). Also seen in SI-Fig. S7 is that the average width of the atomic terraces is about 50 nm, which is the same as the ripple period (see Figs 4d,f). Based on these comparisons, we can make a conclusion that upon laser illuminations, buckling of the SL-MoS_2_ nanosheet, in a similar way as that of ultrathin mica^35^, occurred at the middle of the atomic terraces (see SI, Fig. S6d). Instead of buckling, the SL-MoS_2_ nanosheet was pinned at the step edges of the substrate due to the increased MoS_2_-substrate interactions over there. Most of the individual SL-MoS_2_ ripples are about 1.0 nm in height (see Figs 4d,f). This ripple height is consistent with those observed by transmission-electron microscopy for the exfoliated and spontaneously rippled monolayer MoS_2_^27^,^28^.

**Figure 5:**
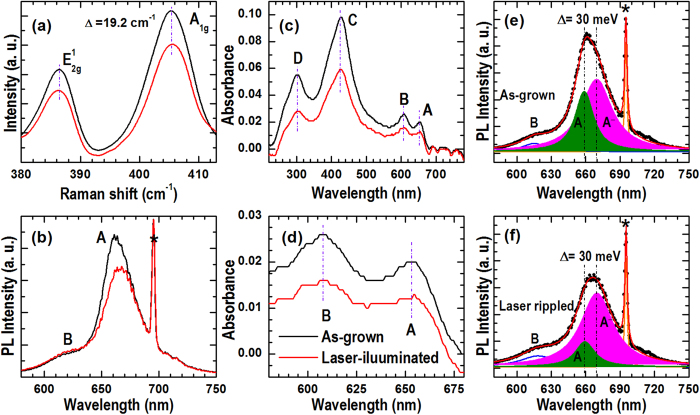
Raman, PL, and absorbance spectra of the SL-MoS_2_ nanosheet before and after the laser illuminations. (**a**) Raman spectra. (**b**) PL spectra. (**c**, **d**) Absorbance spectra. (**e**, **f**) Spectral deconvolutions of the PL emissions. The asterisks indicate the signal from the sapphire substrate.

To study the optical properties of the rippled SL-MoS_2_ nanosheets, we have further carried out laser illuminations (10 × 10 μm^2^ with a step of 0.4 μm) at a position away from the grain boundary. Raman, PL and absorbance spectra were collected before and after the laser illuminations from the center area of the 10 × 10 μm^2^ square. The spectra are shown in Fig. 5. The Raman and the absorbance spectra do not exhibit any distinguishable shifts but an intensity reduction after the laser illumination, indicating a minor strain change induced by the laser illuminations. This is physically reasonable since less than 0.11% of uniaxial tensile strain would be introduced into a cone-wave rippled film with a period of 50 nm and a height of 1.0 nm (see SI, Fig. S8). Phonon shifts caused by such amount of uniaxial tensile strain in SL-MoS_2_ are indeed smaller than 0.2 wavenumbers^36^,^37^. In contrast, a red shift in the A-exciton related PL emissions of about 5.1 nm (i.e., 15 meV) is caused by the laser illumination although its resonant absorption peak does not show any shift (see Figs 5c,d). This red shift in PL is much larger than that could be caused by a uniaxial tensile strain of 0.11% (~5 meV)^38^. However, a careful look at the PL spectra in Fig. 5b revealed that there is absence of peak shift in B-exciton emissions while the red shift in A-exciton emissions is mainly caused by the intensity decrements at its short-wavelength shoulder. In this regard, peak deconvolutions were further carriedout for the PL emissions.

Figures 5e,f present the peak deconvolutions for the PL emissions shown in 5b. One sees that the A-exciton related emissions, before and after the laser-illuminations, are well fitted by two Lorentzian functions. The separation between the two peaks is 30 meV, closely matching the energy difference between the neutral exciton and negatively charged exciton (i.e., trion A^-^) of SL-MoS_2_^39^,^40^. It is also seen that the deconvoluted peaks do not exhibit any shift after the laser illuminations (see Figs 5e,f). This observation is consistent with the Raman and absorbance results (see Figs 5a,c, d), confirming the minor effect of strain. Also seen is that rippling of the SL-MoS_2_ nanosheets induced by the laser-illuminations tends to suppress the neutral exciton recombination but has only a minor effect on the trion emissions. This emission intensity evolution could be related to the spatially separation of photo-carriers into the peak and valley areas as well as the intermediate surface and/or interface environment changes due to the film rippling^26^.

## Discussion

A dispersive growth method has been introduced in conventional CVD growth of SL-MoS_2_ nanosheets on c-plane sapphire substrates with MoO_3_ and S powders as the reaction precursors. Comparisons between the direct and the dispersive growths are schematically shown in SI, Fig. S9. It is generally accepted that in the direct growth, the nucleation and growth of MoS_2_ could occur anywhere on the substrate. The growth is essentially controlled by quite a few parameters. However, with the encapsulation of a shadow mask on the substrate the nucleation of MoS_2_ can only start at the edge of the masked area (see SI, Fig. S9). In this way, the nucleation is spatially controlled and the sensitivity of the growth to the parameters such as the load of MoO_3_ source is largely weakened. The growth is thus more feasibly controlled by the growth temperature and the growth duration. The reaction species are dispersively supplied onto the laterally advancing growth front via diffusion on the grown MoS_2_ layer at elevated temperature (950 °C). The high growth temperature and the nonexistence of dangling bonds on the grown MoS_2_ nanosheets tend to promote the surface mobility of the adatoms towards the growing front under the shadow mask, meanwhile, minimize the nucleation of MoS_2_ on the grown layer. As a result, large-area, continuously, and high quality SL-MoS_2_ nanosheets are strategically obtained. The SL-MoS_2_ nanosheets conformally grown on the c-plane substrate, have a collective in-plan orientation, i.e., MoS_2_[10-10]//Al_2_O_3_[11**-**20]. The boundary area between adjacent SL-MoS_2_ grains exhibits the same blue-shift behaviors in Raman/PL features as those observed previously^24^.

We also demonstrated that regular ripples with the period of 50 nm and the height of 1.0 nm can be introduced into the SL-MoS_2_ nanosheets by laser illuminations. The laser-induced SL-MoS_2_ rippling, via buckling at the middle of the atomic terraces but pinning at the atomic steps, easily initiates from the grain boundaries and extends along the atomic steps of the substrate. The rippling can be associated with a rearrangement of in-plane strains and the interfacial van der Waals interactions between SL-MoS_2_ and the c/6-stepped surface of the c-plane sapphire substrate. Such laser-induced ripple structures, absent in multilayer MoS_2_ nanosheets, only have a minor effect on phonon frequencies and exciton resonance absorption peaks but apparently cause a red shift to the main PL emission peak of the SL-MoS_2_. However, when the main PL emissions are deconvoluted into two peaks (with a separation of 30 meV), corresponding to those of exciton- and trion-dominated emissions, we found that their peak wavelengths are indeed intact after the laser-induced rippling. The relative intensity changes of the exciton- and trion-dominated PL emissions could be related to spatially separations of photo-carriers into the peak and valley areas of the rippled film as well as the changes of the intermediate surface/interface environment caused by the film rippling^26^. The dispersive CVD growth method and the laser-induced rippling phenomenon of high quality SL-MoS_2_ may have important consequences towards wafer-scale large-area single crystal SL-MoS_2_.

## Methods

MoS_2_ atomic layers were grown by CVD using a method similar to ref. 17 but without pre-deposition on the epiready c-plane sapphire substrate. Instead, MoO_3_ powder (30 mg, 99.5%) loaded in a crucible was used as the reaction precursors. The sapphire substrate physically encapsulated by a shadow mask (i.e., a clean Si or quartz disk with the surface roughness of ~5 μm), was set in the downstream at a distance of about 8–10 mm from the MoO_3_ source. The growth was performed at 950 °C for 20 min under atmospheric pressure. Large-area SL-MoS_2_ nanosheets are obtained on the surface of sapphire under the shadow mask, where the growth is named *dispersive* growth (ref. 31 and the references therein) since the substrate is ‘*indirectly*’ exposed to the reaction species.

AFM images were recorded using a tapping mode in a Veeco Dimension-Icon AFM system. Confocal micro-Raman/PL measurements were carried out in a Witec alpha 300 system. The experiments were performed at room temperature in a backscattering configuration using the 532-nm line of an argon-ion laser adjusted to ~5.0 mW as the excitation source. This laser is also used as the illumination source for generating the MoS_2_ ripples simultaneously during the Raman and PL mappings. For the fast Raman and PL mappings (100 × 100 μm^2^), the step size is 4 μm and the integration time is 2.0 s. After this round of mapping, ripples are seen already in the area of grain boundary (see SI, Fig. S5b). For the slow Raman and PL mappings (10 × 10 μm^2^), the step size was reduced to 0.4 μm and the integration time is kept the same (i.e., 2 s). By automatically switching the working mode (controlled by software) between Raman and PL, we are able to collect the typical Raman and PL spectra from exactly the same area of the SL-MoS_2_ nanosheets before and after the laser illuminations, i.e., without and with the laser-induced ripples.

## Additional Information

**How to cite this article**: Liu, H. and Chi, D. Dispersive growth and laser-induced rippling of large-area singlelayer MoS_2_ nanosheets by CVD on c-plane sapphire substrate. *Sci. Rep.* **5**, 11756; doi: 10.1038/srep11756 (2015).

## Supplementary Material

Supplementary Information
