# Supplementary material for: Dispersive growth and laser-induced rippling of large-area singlelayer MoS2 nanosheets by CVD on c-plane sapphire substrate
Source: Sci Rep. 2015 Jun 29;5:11756. doi: 10.1038/srep11756 (PMC4483778; doi:10.1038/srep11756)
Supplement: Supplementary Information [file srep11756-s1.doc]

**Supplementary Information for**

Dispersive growth and laser-induced rippling of large-area singlelayer MoS_2_ nanosheets by CVD on c-plane sapphire substrate

Hongfei Liu[[1]](#footnote-2) and Dongzhi Chi

*Institute of Materials Research and Engineering (IMRE), A*STAR (Agency for Science, Technology and Research), 3 Research Link, Singapore 117602, Singapore*


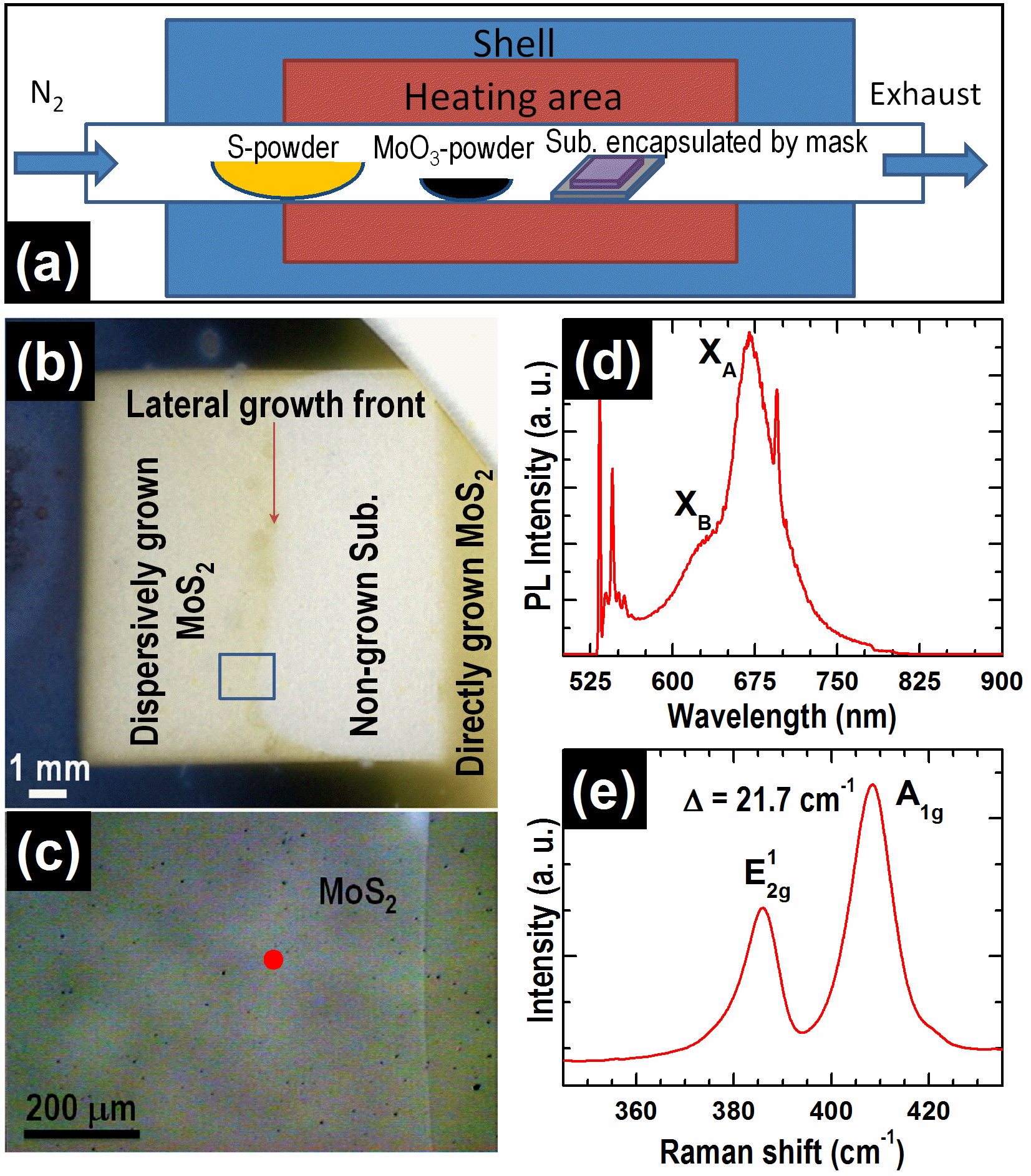


**Figure S1** A typical dispersive growth of large-area singlelayer MoS_2_ nanosheets on c-plane sapphire substrate under a 1 cm × 1 cm Si shadow mask. (a) Schematic diagram of the CVD setup, (b) Optical image shows the lateral growth of nanosheets with the source materials supplied from the left, (c) an enlarge image taken at the growth front (marked in b), (d) and (e) PL and Raman spectra taken around the growth front (marked in c) at room temperature using a 532-nm laser.


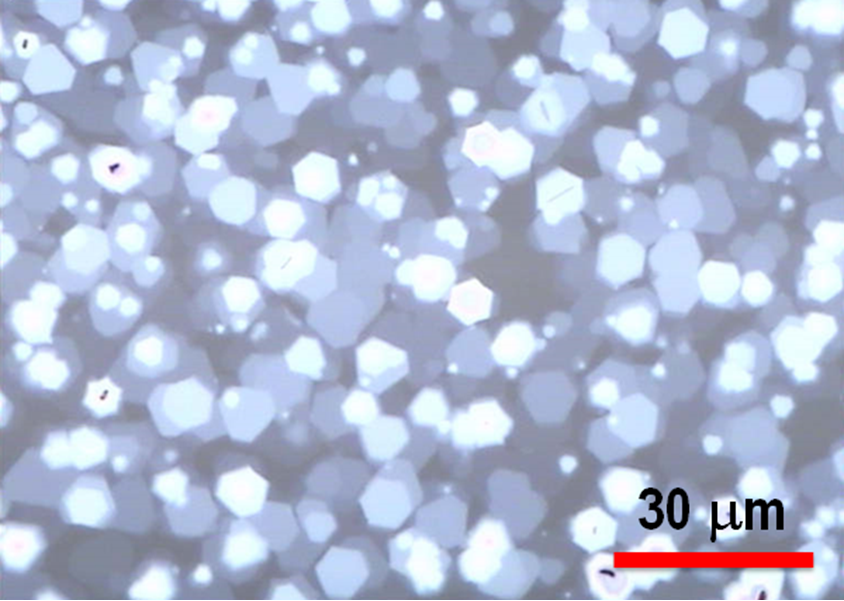


**Figure S2** Optical image showing the triangle and hexagonal MoS_2_ grains in the directly grown sample at the areas a bit far away from the MoO_3_ source.


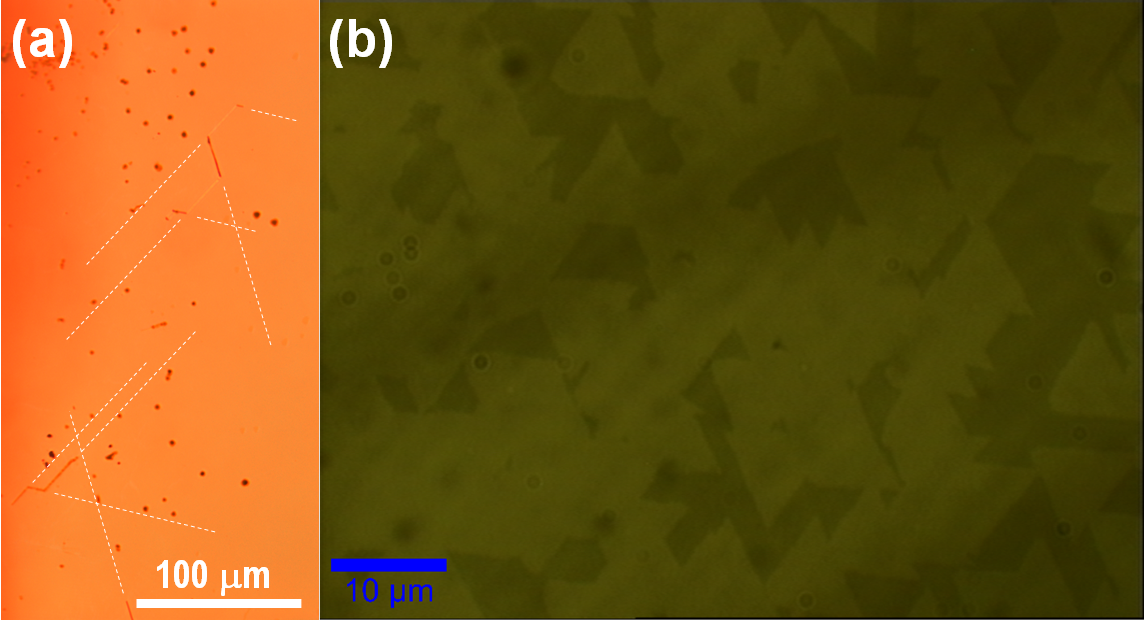


**Figure S3** Optical images (a) showing the 60°-rotated linear structures of a dispersively grown SL-MoS_2_ nanosheets sample (grown at 950 °C) and (b) SL-MoS_2_ grains with parallel edges dispersively grown by CVD at 750 °C

**
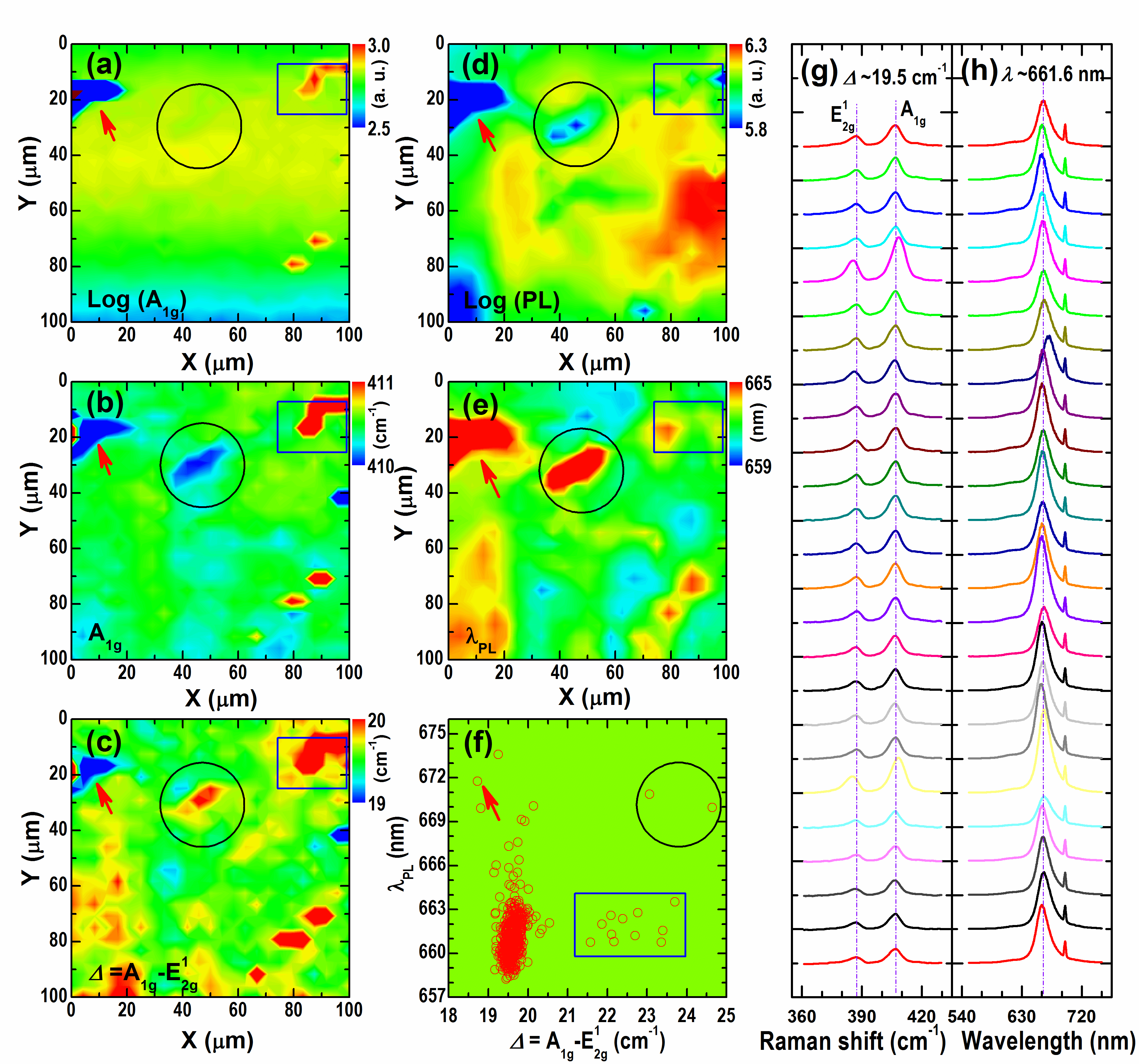
**

**Figure S4** Raman and PL mappings of the dispersively growth MoS_2_ by CVD on c-plane sapphire substrate, both were collected in a mapping step of 4 μm with a 532-nm laser excitation. (a) Intensity in Log scale of A_1g_, (b) Raman shift of A_1g_, (c) Frequency differences between A_1g_ and E_2g_^1^, (d) PL intensity in Log scale, (e) Wavelength of the PL emission peak, (f) Plots of the PL emission peak as a function of the Raman frequency differences generated from the mappings, where 95% of the 625 data fall in the range of Δ= 19.5 ± 0.5 cm^-1^ and λ_PL_= 661.0 ± 2.0 nm. The arrows, circles, rectangles mark the correlations, showing the same area for the Raman and PL mappings. (g, h) Raman and PL spectra exported from the mappings in with a step of 20 μm.


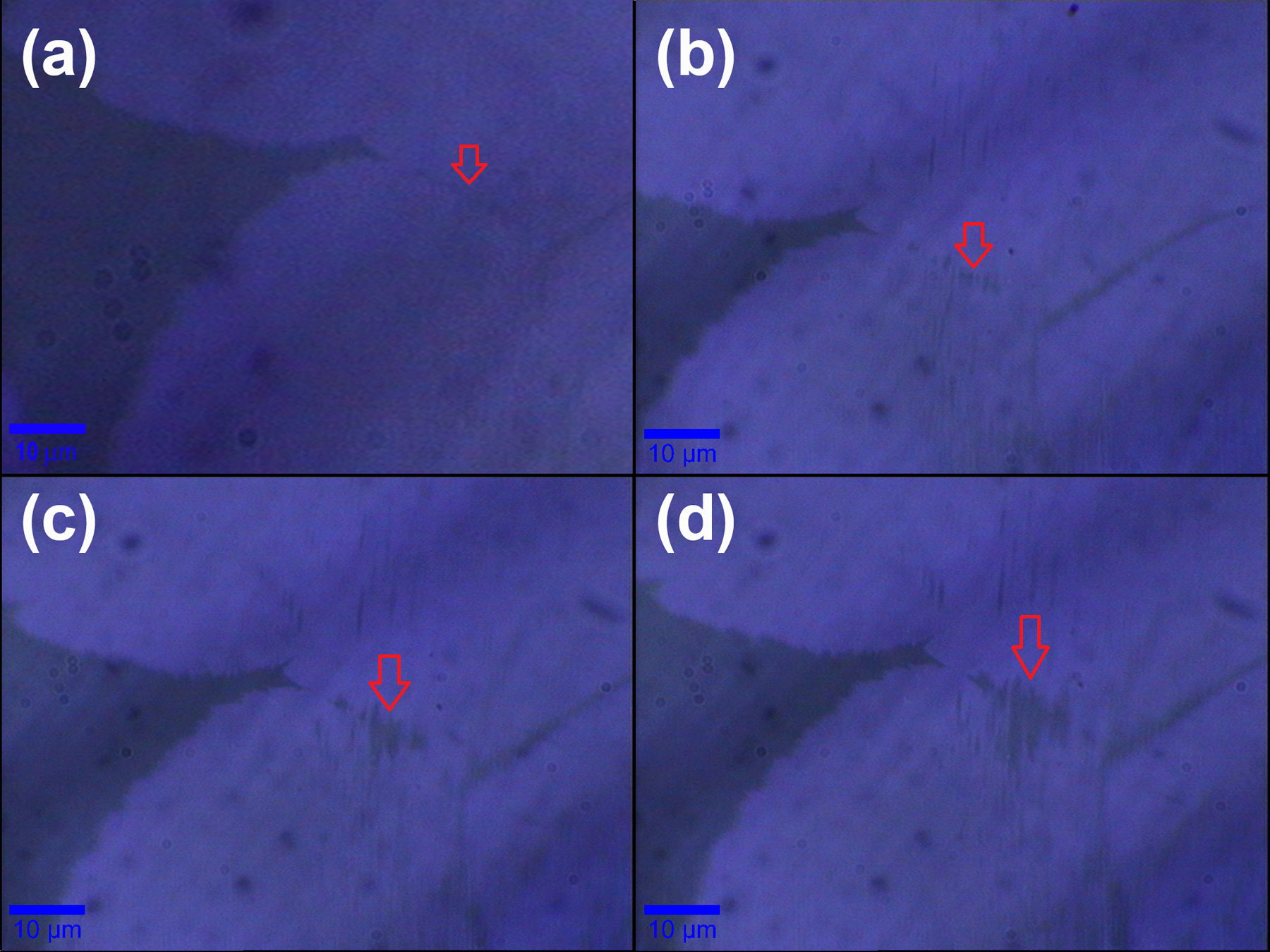


**Figure S5** Optical images showing the morphology evolutions at the grain boundary of the large SL-MoS_2_ nanosheets. (a) Before laser illuminations. (b) After the first round of Raman and PL mappings (100 × 100 μm^2^) with large mapping step (4 μm). (c) After the second round of Raman mapping (10 × 10 μm^2^) with the reduced mapping step (0.4 μm). (d) After the second round of PL mapping (10 × 10 μm^2^) with the reduced mapping step (0.4 μm). The downward arrows indicate the area where the mappings were carried out.


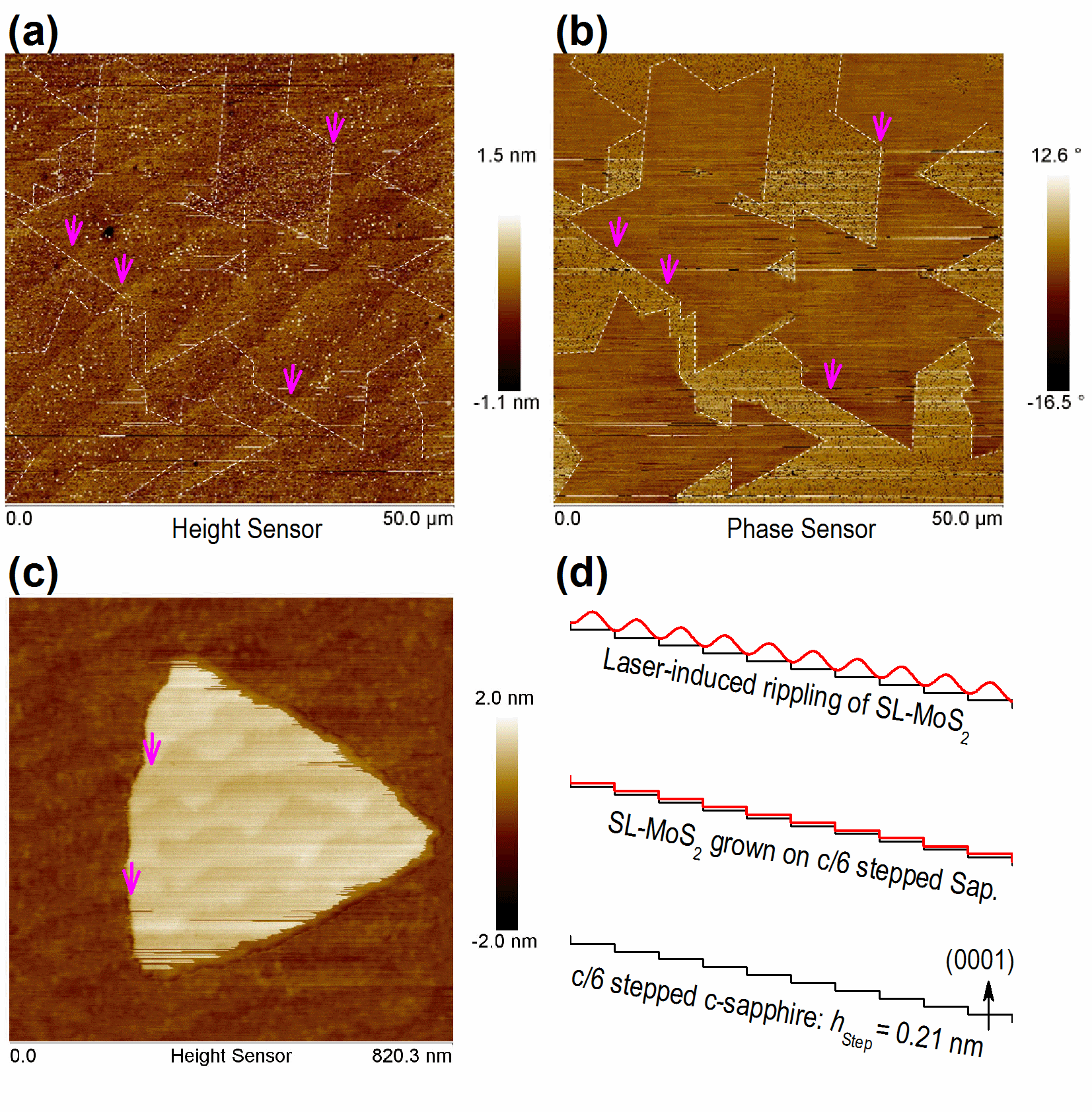


**Figure S6** AFM images of MoS_2_ atomic layers grown on c-plane sapphire substrate. (a) and (b) Height and phase sensors of single-layer (SL) MoS_2_. (c) Height sensor of 2L-MoS_2_. The arrows indicate the continuity of the atomic steps across the MoS_2_ grain edges, showing that the MoS_2_ sheets are conformally grown on the stepped (c/6 = 0.21 nm) surface of the substrate. (d) Summary of SL-MoS_2_ on c/6-stepped c-plane sapphire before and after laser illuminations. Buckling of MoS_2_ occurred at the middle of the atomic terraces; however, the nanosheet is pinned by the atomic steps of the substrate, leading to the regular MoS_2_ ripples.


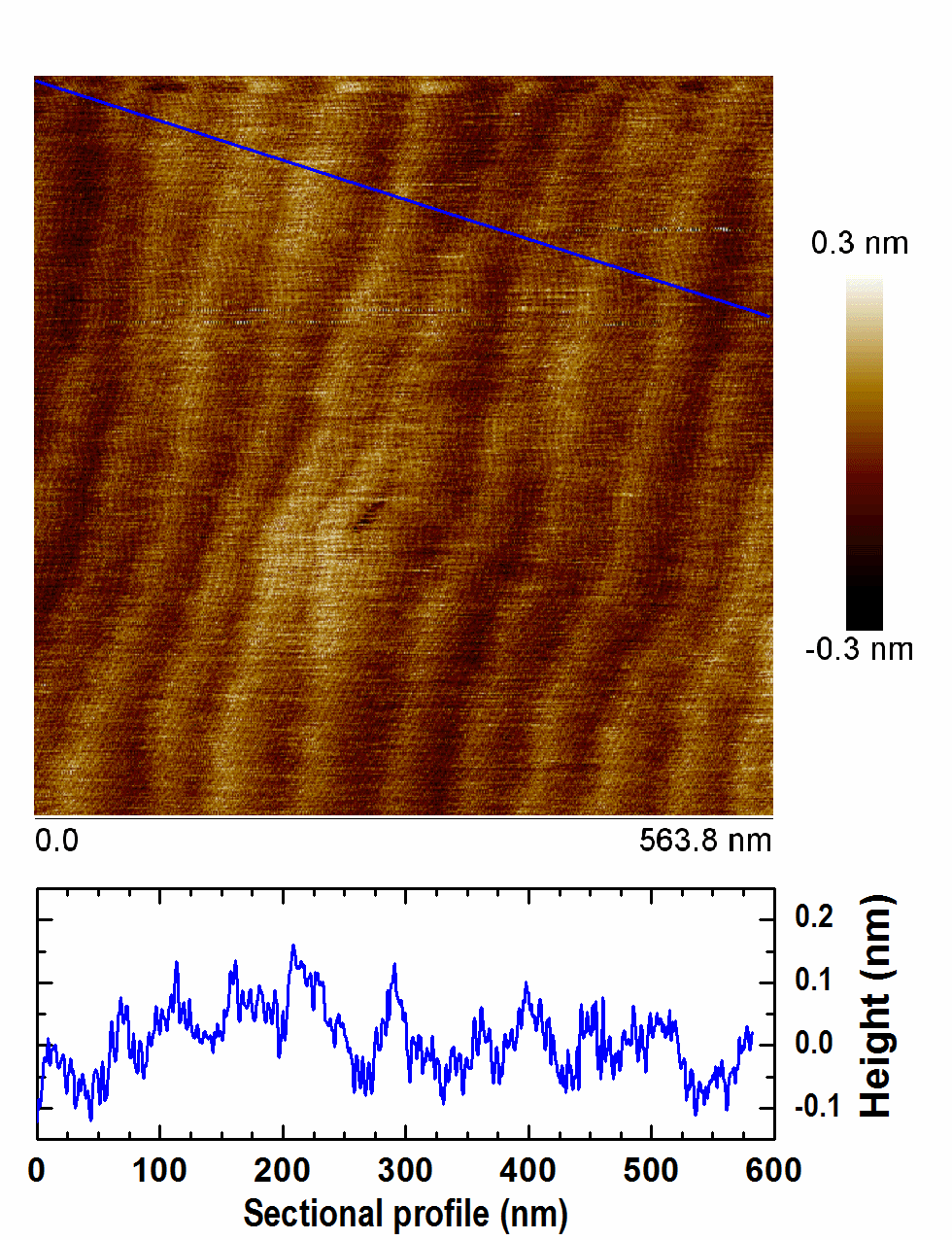


**Figure S7** AFM image taken from an as-grown SL-MoS_2_ nanosheet grown on c-plane sapphire substrate. The average width (~50 nm) of the atomic terraces can be simply calculated from this image. The step heights are generally < 0.2 nm, corresponding to one-sixth (i.e., a mono-step) of the lattice parameter normal to the c-plane of sapphire.[[2]](#endnote-2) This image also shows the conformal growth of MoS_2_ on c/6-stepped c-plane sapphire substrate.


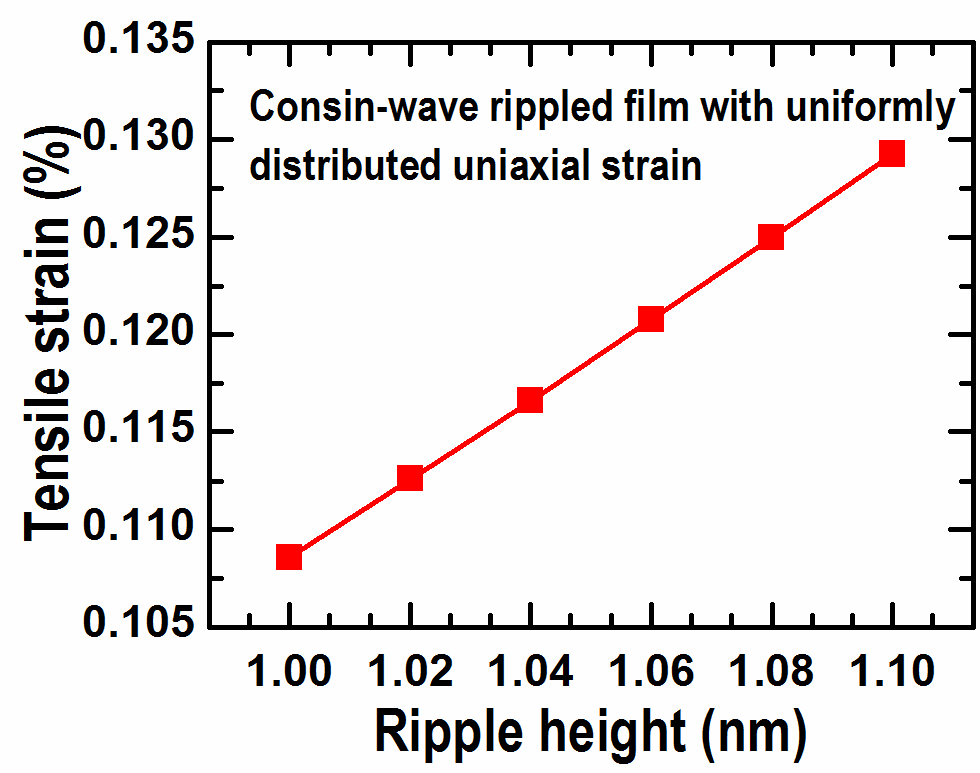


**Figure S8** The strain-height relations for cosine-wave rippled films with λ_Period_ = 50 nm assuming the uniaxial tensile strain is uniformly distributed in the direction perpendicular to the ripple array.


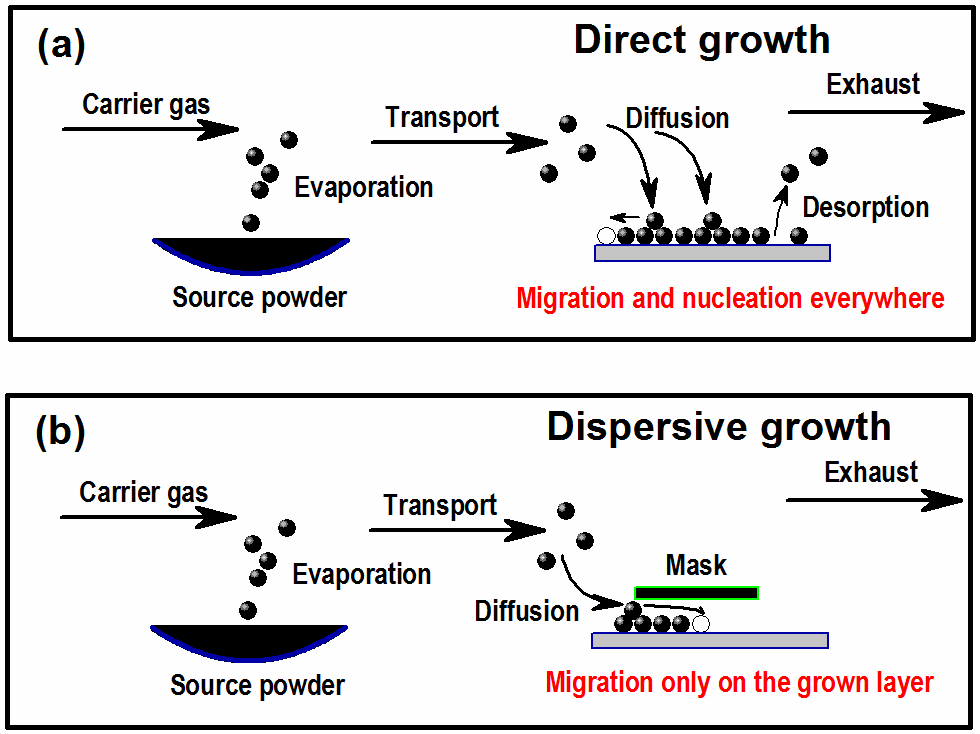


**Figure S9** Schematic diagram showing the difference in nucleation and growth of MoS_2_ via the direct (a) and dispersive (b) methods. In the direct growth the nucleation can occur anywhere on the substrate; however, in the dispersive growth the nucleation can only start at the edge of the masked area. The reaction species are dispersively supplied onto the laterally advancing growth front via diffusion on the grown MoS2 layer at elevated temperature. The laterally advancing growth front under the mask led to the sharp and straight boundary between the grown MoS_2_ nanosheet and the non-grown sapphire surface seen above in Fig. S1b (both area were encapsulated by the mask during the dispersive CVD growth).

References:

1. Corresponding author e-mail: [liuhf@imre.a-star.edu.sg](mailto:liuhf@imre.a-star.edu.sg) [↑](#footnote-ref-2)
2. . Pham Van, L., Kurnosikov, O., Cousty, J. Evolution of steps on vicinal (0001) surfaces of a-alumina, *Surf. Sci.* **411**, 263-271 (1998). [↑](#endnote-ref-2)
